# Spatial Distribution and Antioxidant Activity of Extracts from Citrus Fruits

**DOI:** 10.3390/antiox12040781

**Published:** 2023-03-23

**Authors:** María García-Nicolás, Carlos A. Ledesma-Escobar, Feliciano Priego-Capote

**Affiliations:** 1 Department of Analytical Chemistry, Faculty of Chemistry, Regional Campus of International Excellence “Campus Mare Nostrum”, University of Murcia, E-30100 Murcia, Spain; 2 Department of Analytical Chemistry, Annex Marie Curie Building, Campus of Rabanales, University of Córdoba, E-14014 Córdoba, Spain; 3 Maimónides Institute of Biomedical Research (IMIBIC), Reina Sofía University Hospital, University of Córdoba, 14004 Córdoba, Spain; 4 Nanochemistry University Institute (IUNAN), Campus of Rabanales, University of Córdoba, E-14014 Córdoba, Spain; 5 CIBER Fragilidad y Envejecimiento Saludable (CIBERFES), Instituto de Salud Carlos III, 28029 Madrid, Spain

**Keywords:** citrus fruits, metabolomics, spatial distribution, antioxidant activity, flavonoids, limonoids, carboxylic acids

## Abstract

Citrus fruits are recommended components of the human diet because of their enriched composition in bioactive compounds and health benefits. Among their notable components are phenols, with a special emphasis on flavonoids, limonoids, and carboxylic acids. In this research, we have carried out a spatial metabolomics analysis for the characterization of these bioactive families in three citrus fruits, namely, lemons, limes, and mandarins. Sampling was undertaken, for which the juices and three fruit tissues, namely, albedo, flavedo, and segments, were analyzed. This characterization allowed for the determination of 49 bioactive compounds in all the samples. The composition of the different extracts was correlated with the antioxidant capacity measured by the DPPH radical scavenging activity and β-carotene bleaching assays. Flavonoids, found in the albedo and flavedo at higher concentrations, were the main components responsible for DPPH radical scavenging activity. On the other hand, the combined action of flavonoids and limonoids contributed to explaining the antioxidant activity measured by the β-carotene bleaching assay. Generally, the antioxidant capacity of juices was lower than that estimated for extracts from citrus tissues.

## 1. Introduction

Citrus fruits constitute a characteristic element of the Mediterranean diet and are extensively researched due to their bioactive composition and health benefits. Among the most known citrus fruits, lemons (Citrus limon), mandarins (Citrus reticulata), and Persian limes (Citrus latifolia) have been demonstrated to be outstanding sources of bioactive compounds such as ascorbic acid, limonoids, and flavonoids [1].

Flavonoids are the most predominant phenolic compounds in citrus fruits and are the main sources of their flavor and coloration [2]. These phytochemicals have been described as beneficial to human health and are used to prevent some diseases [3] because of their anti-inflammatory, anti-cancer, or anti-obesity properties [4,5,6,7]. Citrus flavonoids’ phenolic activities, such as their free radical scavenging activity, pro-oxidant metal ion chelation, and ability to act as enzyme cofactors, explain their beneficial properties.

Limonoids have also received great attention because of their anti-inflammatory, anti-aging, anti-tumor, immunomodulatory, and antioxidant activity [8]. Limonoids can be found as glucosides (water soluble) or aglycones (water insoluble), which contribute to the unflavored or bitter taste of citrus fruits. In a recent study, a total of 18 limonoid glucosides and 55 limonoid aglycones were reported in citrus fruits [9]. The most representative limonoid glucoside is limonin-17-β-D-glucopyranoside, while the most abundant aglycones are limonin and nomilin [10,11]. The presence of limonoids has mainly been detected in essential oils obtained from citrus flavedo, and these oils have received special attention in previous years because of their high antimicrobial and preservative activity in various foods [12,13,14].

Limonoids and flavonoids have been determined by different techniques, with the most extensive being liquid chromatography coupled with diode array detection (LC–DAD) [15]; however, this technique requires the use of analytical standards for identification. For this reason, in the few last years, the use of liquid chromatography coupled with high-resolution tandem mass spectrometry (LC–MS/HRMS) has been increasing. Although this technique also requires standards for confirmation, it provides better identification capacity by comparing MS2 spectra with those included in databases. Most citrus fruits have been characterized by this technique, for example, limes, oranges, tangerines, lemons, grapefruit, etc. [16,17,18,19,20,21,22].

The antioxidant properties of citrus limonoids and flavonoids have been previously described using various techniques, including 2,2-diphenyl-1-picrylhydrazyl (DPPH) antioxidant capacity and β-carotene blenching assay studies. The DPPH method relies on the strength of a 30 min radical scavenging time, which allows DPPH to react efficiently, even with weak antioxidants providing high sensitivity, while β-carotene test allows both lipophilic and hydrophilic samples to be analyzed with high reproducibility [23]. The combination of these two assays has been used for the determination of the antioxidant activity of limonoid and flavonoid extracts from different tissues [17,24]. Specifically, grapefruit seeds [25], *Rutaceae* species peel [26], albedo and flavedo from *pompia* fruits [16], kinnow peel [27], and Spanish clementine fresh pulp [28] have been analyzed for the presence of these high valuable compounds using both antioxidant determination assays. Moreover, changes in the limonin and nomilin concentrations in different tissues from pummelo and mandarin varieties have been studied during fruit growth and maturation [29]. However, although citrus antioxidant activity has been widely studied, to the best of our knowledge, the spatial distribution of the antioxidant capacity in the different tissues (flavedo, albedo, pulp segments, and juice) of citrus fruits and the synergistic interaction between limonoids and flavonoids have not been studied in detail. For this reason, the aim of this research was to characterize the limonoid and flavonoid fractions of three Citrus fruits, namely, lemons, limes, and mandarins. For this purpose, three main tissues (albedo, flavedo, and pulp segment) and their respective juices were studied. A highly selective and sensitive LC–MS/HRMS approach was used for the characterization of these citrus tissues. In addition, their antioxidant activity was evaluated through the DPPH radical scavenging activity and β-carotene bleaching assays to ascertain the properties of limonoids and flavonoids and elucidate any synergistic interactions.

## 2. Materials and Methods

### 2.1. Samples

Limes, mandarins, and lemons were purchased in two local markets in Cordoba and Murcia (La Corredera and Veronicas local markets, Spain) in October 2022. Fruits were washed and processed to separate the albedo, flavedo, pulp segments, and juice. The solid samples were lyophilized and subsequently ground. The powder was stored in the dark at −20 °C.

### 2.2. Reagents

Ethanol (EtOH), methanol (MeOH), ethyl acetate (EA), acetone, chloroform, acetonitrile (ACN), and formic acid were purchased from Scharlab (Barcelona, Spain). n-Hexane was obtained from Sigma−Aldrich (St. Louis, MO, USA). All solvents were LC- or MS-grade. Simplicity^®^ UV Millipore equipment (Burlington, MA, USA) was used to generate purified Milli-Q-water. Syringaldehyde (SA), used as external standard, was acquired from Sigma−Aldrich. For antioxidant assays, we used 1,1-diphenyl-2-picrylhydrazyl (DPPH), linoleic acid, Tween-20, and β-carotene standards obtained from Sigma-Aldrich. Silica gel 60 (0.06–0.2 mm particle size) from Merck (Darmstadt, Germany) was used for the fractionation of extracts.

### 2.3. Instrumentation and Software

The analytical equipment consisted of a 1200 series LC system from Agilent Technologies (Palo Alto, CA, USA) coupled with a dual electrospray ionization source and a high-resolution Agilent 6540 quadrupole–time of flight detector QTOF (LC–MS/HRMS).

Agilent MassHunter Workstation (version B6.00 Profinder, Agilent Technologies, Santa Clara, CA, USA) was used for data acquisition. MassHunter Qualitative v7.0 software and MetaboAnalyst 5.0 (https://metaboanalyst.ca/, accessed on 9 January 2023) were used for targeted extraction of MS/MS information, metabolomics data analysis, and interpretation.

### 2.4. Metabolites Extraction

Albedo, flavedo, and pulp segments of mandarins, limes, and lemons were crushed and homogenized after lyophilization. A total of 1 g of each tissue was extracted in 10 mL of n-hexane while stirring at 1000 rpm for 30 min at 30 °C to remove the lipidic fraction. The n-hexane extract was discarded, and the solids were recovered and dried under gentle nitrogen flow prior to the triplicate extraction of bioactive compounds with 10 mL of (50:25:25 *v*/*v*/*v*) EA/acetone/EtOH for 30 min at 30 °C. The three fractions were mixed, evaporated under vacuum, and then reconstituted in 1 mL of MeOH containing SA at 1 µg/mL. Six extracts were obtained from each tissue (three extracts for triplicate analysis of the antioxidant activity and three extracts for fractionation).

Lyophilized juice samples of the three fruits were solubilized in water at 150 mg of total solids/mL. A total of 10 mL of reconstituted juice was extracted with 5 mL of EA for 10 min at 30 °C. The EA fraction was dried under vacuum and reconstituted in 1 mL of MeOH with SA at 1 µg/mL.

### 2.5. LC–MS/HRMS Analysis

Chromatographic separation of the extracts was performed by using a Zorbax Eclipse Plus C18 chromatographic column (1.8 μm particle size, 150 × 3.0 mm i.d., Agilent Technologies). The injection volume was 2 μL, and the mobile phases were composed of 0.1% of formic acid in both deionized water (phase A) and acetonitrile (phase B). Flow rate was constant at 0.25 mL/min. Chromatographic gradient was programmed as follows: 4% phase B was selected as initial composition; then, from min 0 to 1, mobile phase B was increased to 25%; from min 1 to 6, phase B was changed to 40%; from min 6 to 8, phase B was modified to 60%; and from min 8 to 10, mobile phase B was increased from 60 to 100%. The latter composition was maintained for 12 min to guarantee the elution of metabolites and the sterilization of the column. A post-time of 13 min was set to equilibrate the initial conditions for the next analysis.

The dual electrospray ionization source (ESI) was operated in both positive and negative ionization modes. ESI parameters were as follows: nebulizer gas at 50 psi and drying gas flow rate and temperature at 10 L/min and 325 °C, respectively. The capillary voltage was set at 3500 V, while the fragmentor, skimmer, and octapole voltages were 130, 65, and 750 V, respectively. Data were acquired in centroid mode in the extended dynamic range (2 GHz). Full scan was carried out at 6 spectra/s within the *m*/*z* range of 60–1200, with subsequent activation of the three most intense precursor ions (allowed charge: single or double) by MS/MS using a collision energy of 20 eV and 40 eV at 3 spectra/s within the *m*/*z* range of 30–1200. An active exclusion window was programmed for 0.75 min to avoid repetitive fragmentation of the most intense precursor ions, thus increasing the detection coverage. To assure the desired mass accuracy of recorded ions, continuous internal calibration was performed during analyses with the use of signals at *m*/*z* 121.0509 (protonated purine) and *m*/*z* 922.0098 (protonated hexakis (1H, 1H, 3H-tetrafluoropropoxy) phosphazene or HP-921) in the positive ionization mode and *m*/*z* of 112.9856 (trifluoroacetic acid anion) and *m*/*z* 1033.9881 (HP-921) in the negative ionization mode. A quality control sample (QC) obtained by mixing aliquots of extracts was used to ensure the reproducibility and robustness of the methodology and chemometric results.

### 2.6. Data processing and Statistical Analysis

MassHunter Profinder (version B10.00; Agilent Technologies, Santa Clara, CA, USA) was used to process the data obtained by LC–MS/HRMS. Treatment of the raw data file started with the extraction of potential molecular features (MFs) by the applicable algorithm included in the software. The recursive extraction algorithm considered all ions exceeding 5000 counts as cut-off value. Additionally, the isotopic distribution used to determine whether a molecular feature is valid should be defined by two or more ions (with a peak spacing tolerance of *m*/*z* 0.0025 plus 10.0 ppm in mass accuracy). Apart from [M + H]^+^ and [M−H]^−^ ions, adduct formation in the positive (Na^+^) and negative ionization (HCOO^−^, Cl^−^) modes and neutral loss by dehydration were included to identify features corresponding to the same potential metabolite. Thus, ions with identical elution profiles and related *m/z* values (representing different adducts or isotopes of the same compound) were extracted as entities characterized by their retention time (RT), the intensity at the apex chromatographic peak, and accurate mass. Then, a recursion step assured correct integration of the entities in all analyses.

Identification was achieved following the methodology previously described by Ledesma-Escobar et al. [17]. After extraction and alignment of all MFs, the software MassHunter Qualitative v.7.0 was used for targeted extraction of MS2 information associated with the monitored MFs in the whole dataset. This information was used for tentative identification of metabolites by searching the MoNA-MassBank of North America (https://mona.fiehnlab.ucdavis.edu/spectra/search, accessed on 13 December 2022) database and others developed by the research group. Additionally, some compounds were confirmed according to both their MS2 information and retention time by using commercially available standards. Finally, the compounds that were not found in the databases, or whose commercial standards were unavailable, were identified via an analysis of neutral mass losses combined with the characteristic fragmentation patterns of their derivatives confirmed by commercial standards.

Quantitative analysis was carried out in relative terms by preparing calibration models for limonin, nomilin, hesperidin, and quercetin as standard compounds (determination coefficients > 0.98; calibration range between 50 ng/mL and 20 μg/mL). Syringaldehyde (1 μg/mL) was used as external standard. Flavonoid glucosides and aglycones were quantitated with models prepared with hesperidin or quercetin, respectively, while limonoids were determined using their corresponding aglycone standards.

### 2.7. Silica Gel Column Fractionation of Crude Extracts

Crude extracts (1 mL) were fractioned by being passed through a normal-phase silica gel (5 g) column. The solid phase was conditioned with hexane and the metabolites were eluted with 2 mL of EA (fraction 1), 2 mL of 1:1 (*v*/*v*) EtOH/acetone (fraction 2), and 2 mL of 1:1 (*v*/*v*) EtOH/H_2_O (fraction 3). Each fraction was evaporated under vacuum and dissolved with 1 mL of MeOH. The solutions were stored at −80 °C in darkness until use.

### 2.8. Antioxidant Assays

#### 2.8.1. DPPH Radical Scavenging Activity

DPPH radical scavenging activity was evaluated by mixing 100 µL of sample (either crude extract or fraction) with 900 µL of 20 mg/L DPPH solution in MeOH. The mixture was stirred for 30 min at room temperature and the absorbance was measured at 517 nm. Reference solution was prepared following the same procedure but using MeOH instead of sample. The antiradical scavenging potential was expressed as the percentage of decoloration of DPPH solution according to the following equation:

(1) $$\%Radical\;scavenging=\left[1-\frac{A_{s}}{A_{R}}\right]*100$$

where: *As*, absorbance of the sample; *A_R_*, absorbance of the reference solution.

#### 2.8.2. β-Carotene Bleaching Assay

A stock solution for this test was prepared as follows: 500 µL of Tween-20 was dissolved in 50 mL of water previously saturated with O_2_ by air bubbling and mixed with 25 µL of linoleic acid and 500 µL of 1 mg/mL β-carotene solution in chloroform. The mixture was subjected to rotary evaporator under reduced pressure at 30 °C to remove chloroform. Then, the mixture was shaken vigorously until a crystalline emulsion was obtained (approximately 5 min) with the characteristic orange color of β-carotene. Stock solution was immediately used after preparation.

For the measurement of antioxidant activity, 100 µL of the crude extract or fraction was added to 900 µL of the β-carotene/linoleic acid stock solution and stirred for 2 h at 50 °C. The absorbance of the samples was measured at 470 nm immediately after preparation (time 0 min) and after 2 h (time 120 min). A control sample was prepared with MeOH instead of sample. The blank was composed of water, Tween-20, and linoleic acid at the same proportions as those used in the stock solution. The antioxidant activity was calculated as the ability to inhibit the discoloration of β-carotene using the following equation:

(2) $$\%Antioxidant\;activity=\left[1-\frac{\left(A_{s}^{0}-A_{s}^{120}\right)}{\left(A_{c}^{0}-A_{c}^{120}\right)}\right]*100$$

where: *As*, Absorbance of the sample; *Ac*, absorbance of control; 0 and 120 refer to time (min).

## 3. Results

### 3.1. Characterization of Bioactive Compounds in Citrus Fruits

The analysis of the sample citrus extracts led to the identification of 49 compounds distributed in 31 flavonoids, 9 limonoids, 6 simple phenols, and 3 carboxylic acids by following the methodology previously described by the authors [17]. Thirty-two metabolites were successfully identified as confirmed by the analytical standards. In addition, six isomers (MS2 spectrum correlation with that of the standard above 0.9) were detected at different retention times. The rest of the 11 metabolites were tentatively identified via the detection of neutral mass losses and fragmentation patterns. Thus, for the tentative identification of flavonoid derivatives, the MS2 spectrum should include the product ion of the aglycone as the dominating peak and the neutral loss of the distinctive glucoside. Concerning limonoids, their product ion at *m*/*z* 161.0595 in the positive ionization mode dominated the MS2 spectra together with the precursor ion. The identification parameters are summarized in Table S1.

### 3.2. Locations of the Bioactive Compounds in Citrus Fruits

**Figure 1:**
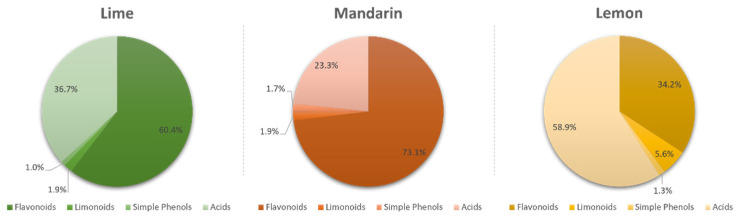
Proportion of flavonoids, limonoids, simple phenols, and carboxylic acids in limes, mandarins, and lemons.

Flavonoids were the most abundant phenolic compounds among the studied metabolites, followed by limonoids, simple phenols, and carboxylic acids. However, each metabolite class was distributed in a different proportion depending on the fruit. Figure 1 shows that flavonoids constituted the most abundant bioactive fraction in limes and mandarins, representing 64.4% and 73.1% of the total content of identified compounds, respectively. In addition, carboxylic acids were the second largest group in both fruits (36.7% in limes and 23.3% in mandarins). However, acids predominated in lemons, constituting 58.9% of the total quantified compounds, followed by flavonoids (34.2%), limonoids (5.6%), and simple phenols (1.3%).

**Figure 2:**
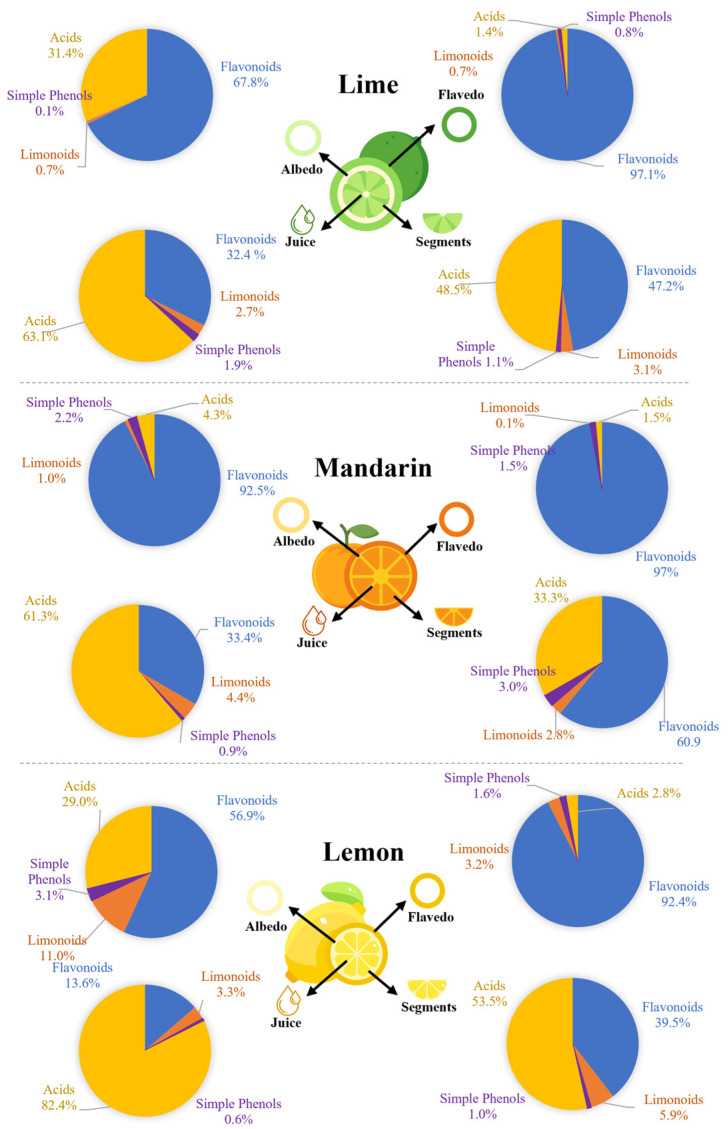
Spatial distribution of flavonoids, limonoids, simple phenols, and carboxylic acids in citrus fruits.

A spatial analysis was carried out to determine the distribution of these metabolites in the analyzed fractions (flavedo, albedo, segments, and juice). Generally, we observed that flavonoids were mostly located in the flavedo and albedo, while carboxylic acids were abundant in the segments and juice. Regarding limonoids, they were mostly found in albedo and segments, while simple phenols were equally distributed in all parts. Despite the similar distribution of the monitored families in the analyzed samples, our results revealed that the fruit was also determinant (Figure 2).

Finally, the concentration of simple phenols was mainly represented by pyrogallol among the three studied fruits. The concentrations of individual metabolites can be found in Table S2. Calibration curves were obtained for limonin, nomilin, hesperidin, and quercetin, which were used also to quantify the rest of the flavonoid glucosides, aglycones, and limonoids.

**Figure 3:**
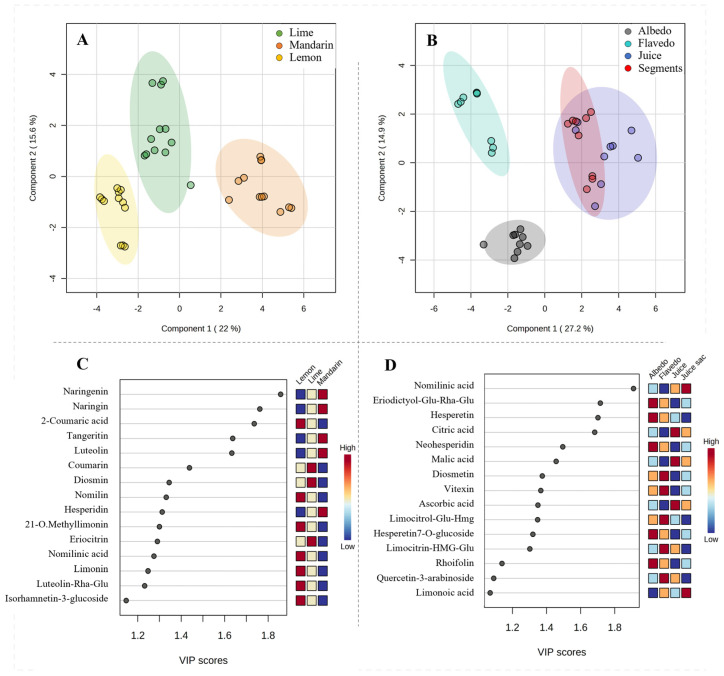
Multivariate PLS-DA for discrimination of citrus fruits (**A**) and samples (**B**) based on the composition of extracts. Variable importance projection (VIP) scores for identification of metabolites with the highest discrimination capability of citrus fruits (**C**) and samples (**D**).

To confirm the observed differences among the fruits and samples, a non-supervised principal component analysis (PCA) was conducted by grouping the matrix by fruit or by sample, including the QCs (Supplementary Figure S1). This analysis revealed clear discrimination in the 3D scores plot for both grouping cases. Once clustering was confirmed, a multivariate analysis by partial least squares discriminant analysis (PLS-DA) was applied by (i) grouping the matrix by fruit (Figure 3A) and (ii) by sample (Figure 3B). The results of these two analyses revealed that the most important variables distinguishing the fruits were flavonoids and coumaric acid (especially naringenin derivatives, tangeritin, and luteolin, Figure 3C). On the other hand, discrimination by samples was mainly explained by limonoid and carboxylic acid concentrations (Figure 3D).

### 3.3. Antioxidant Activity of Extracts from Different Citrus Fruit Tissues

**Figure 4:**
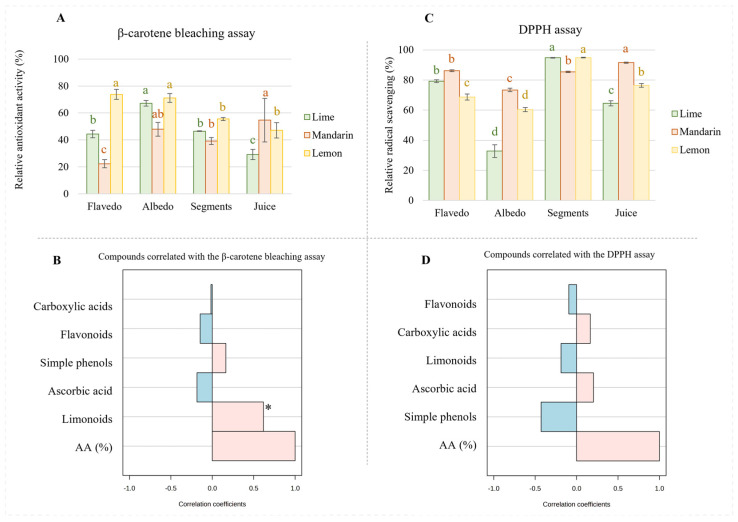
Antioxidant activity (**A**) and radical scavenging capacity (**B**) measured in extracts from citrus fruits. Spearman correlation between antioxidant assays and concentrations of families of metabolites (β-carotene test, (**C**); DPPH assay, (**D**)). Different letters for the same fruit indicate significant differences among tissues extracts in terms of antioxidant or radical scavenging activity. * *p*-value < 0.001.

Concerning the antioxidant activity measured by the β-carotene assay (Figure 4A), our results revealed that, generally, the lemon extracts from the flavedo, albedo, and segments possessed the highest antioxidant activity followed by those from limes and mandarins; however, it was observed that mandarin juice has superior activity compared to lemon and lime juices. Considering the composition of the extracts, it is possible to assume that limonoids play a key role with respect to antioxidant capacity since this class of metabolites is particularly concentrated in the extracts from the flavedo, albedo, and segments of lemons as well as in mandarin juice. Moreover, a Spearman correlation supported this assumption for limonoids (Figure 4B). The mechanism of the β-carotene bleaching assay supports the ability of bioactive compounds to minimize the peroxidation of linoleic acid, given that hydroperoxides from linoleic acid can react with β-carotene. Therefore, greater activities determined by this assay indicate a higher capacity to prevent the oxidation of lipids.

On the other hand, the DPPH assay is based on the ability of bioactive compounds to stabilize free radicals by donating protons. In this sense, this study revealed that the mandarin extracts obtained from flavedo had higher radical scavenging potential than those from limes and lemons (Figure 4C), which could be attributed to a higher proportion of flavonoids (see Figure 2A–C). This assumption is not supported by the analysis of the radical scavenging activity in other samples. Thus, the mandarin extracts obtained from albedo, which were highly enriched in flavonoids, presented greater antioxidant activity than lemons, even when their content in flavonoids was very similar. On the other hand, the lime extracts exhibited almost half of the radical scavenging potential of lemons or mandarins, whose concentrations of flavonoids were only 20%, approximately. These discrepancies were also observed in the segment extracts that were expected to provide the highest radical scavenging activity due to their flavonoid content. Particularly, lemon and lime extracts, which proportionally had lower content in flavonoids than mandarins, provided greater scavenging potential. Finally, similar inconsistencies were observed regarding lemon juice, for which higher scavenging activity was reported compared to that of limes despite the reduced content of flavonoids in the former. According to the extracts’ compositions, it seems that the content of carboxylic acids should have affected antioxidant capacity. In fact, the Spearman correlation revealed that acids, especially ascorbic acid, greatly influence radical scavenging potential despite the content of flavonoids (Figure 4D).

### 3.4. Antioxidant Activity of Three Fractions of the Extracts from Different Citrus Fruits

**Figure 5:**
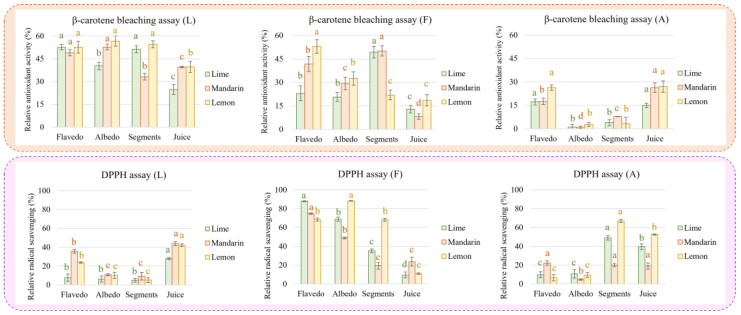
Antioxidant activity (β-carotene bleaching assay) and radical scavenging potential (DDPH assay) measured in fractions enriched in limonoids (L), flavonoids (F), and carboxylic acids (A) obtained by separation of extracts from citrus fruits. Different letters for the same fruit and fraction indicate significant differences in antioxidant or radical scavenging activity.

To disclose the observed complexity of the studied fruits’ antioxidant activity and radical scavenging potential, the extracts were partially purified by using a chromatographic column packed with silica. The purification of complex mixtures such as citrus extracts is not simple; however, three defined fractions were collected: (i) the less polar fraction (L) (with high content of limonin and nomilin), the major limonoids in the extracts, aglycone flavonoids, the minor flavonoids, and a low concentration of conjugated flavonoids; (ii) the mid-polar fraction (F), which is mainly composed of major flavonoids, simple phenols, and, to a lesser extent, conjugated limonoids (minor fraction); and, finally, (iii) the polar fraction, (A) containing mainly carboxylic acids and small amounts of flavonoids and simple phenols (Supplementary Figure S1). As expected, the activity of the three fractions was lower than that of the unfractionated extracts. Nevertheless, the obtained results suggest a synergic effect since the addition of the activities of the three fractions resulted in a higher value than those observed in the crude extracts (Figure 5).

## 4. Discussion

In this report, the spatial distribution and antioxidant activity of bioactive compounds in limes, lemons, and mandarin tissues were studied. We observed that flavonoids were predominant in the flavedo and albedo of the three fruits (97.1% and 67.8%, respectively, in limes; 97.0% and 92.5% in mandarins; and 92.4% and 56.9% in lemons). However, hesperidin, diosmin, and eriocitrin were the most concentrated flavonoids in limes; hesperidin and naringin were predominant in mandarins; and apigenin and diosmetin derivatives and hesperidin were highly concentrated in lemons. The presence of these functional ingredients has been previously described in other citrus limon varieties but not distributed in the different tissues of the fruit [16]. Similarly, limonin derivatives were the most concentrated limonoids in limes and mandarins, while nomilin derivatives were the most abundant in lemons.

The highest proportion of carboxylic acids was found in lemons, followed by mandarins and limes, with citric acid being the most concentrated in all cases, followed by malic and ascorbic acids. However, it is worth noting that lemons were the richest fruit in terms of carboxylic acids (Figure 1), especially ascorbic acid, which was found at a twofold higher concentration in lemons than in limes and mandarins. The presence of the identified carboxylic acids along with others was examined in lemons prior to this study and thus confirms this statement [17].

The antioxidant activity and radical scavenging capacity of citrus fruits have been widely discussed in the literature, but most studies have targeted two main sources: juice and industrial residue extracts [24]. Generally, citrus fruit’s antioxidant activity is mainly attributed to ascorbic acid, phenols, and, to a lesser extent, other minor compounds such as limonoids [24]. Measuring the antioxidant activity of complex mixtures such as citrus juices or extracts is not an easy task since this act is affected by diverse aspects such as the structural [30], pH [31], or synergistic effects precipitated by different chemical families [32,33]. In this study, we evaluated the antioxidant activity in extracts from different citrus fruits to discern the contribution of the studied compounds to bioactivity. For this purpose, we used the β-carotene bleaching assay and the radical scavenging potential test (DPPH assay).

The β-carotene assay, which accounted for the composition of the extracts, showed that limonoids are crucial for antioxidant capacity given that this class of metabolites is particularly concentrated in mandarin juice and in the extracts from flavedo, albedo, and segments of lemons. Our DPPH assay revealed that despite their flavonoid content, carboxylic acids are believed to influence antioxidant capacity. Altunkaya et al. [31] demonstrated that the antioxidant activity of lettuce extracts also depended on the pH and level of synergism with added phenols. Hence, the content of carboxylic acids alters the total antioxidant activity of extracts.

Finally, the purification of the extracts was carried out using a column packed with silica defining three fractions (less, mid, and polar). Concerning the limonoid fraction (L), we observed that the antioxidant activity was like that of the extract, with only an approximate decrease of 20% for all samples. This result reinforces the hypothesis that limonoids play a key role in reducing the formation of hydroperoxides from linoleic acid and thus lipidic peroxidation. In addition, the antioxidant activity was generally lower in the juices compared to the extracts from fruit tissues. On the contrary, the radical scavenging activity in juice was reduced by more than 50% compared to the extract. In this case, juices demonstrated higher capacity than the extracts from fruit tissues. These results agree with those observed by Yu et al. [25], who showed that limonin had reduced radical scavenging capacity. On the other hand, the fractions rich in flavonoids (F) presented an antioxidant activity around 30% lower than that measured in the extracts, with the highest levels observed in lemon flavedo and mandarin and lime segments. Complementarily, the radical scavenging potential of this fraction was only reduced by around 10%. The maximum DPPH activity was found in flavedo from limes and mandarins and in lemon albedo, while minimum values were measured in juices. Finally, the carboxylic acids fraction (A) was particularly affected with respect to antioxidant capacity as determined in both assays. Thus, juice provided better values than those found in fruit tissues except for lime and lemon segments, which presented an antioxidant capacity similar to that obtained from juice.

## 5. Conclusions

This study revealed that the spatial distribution of bioactive compounds is constant in the three citrus fruits. Thus, flavonoids are mostly located in the citrus peel (in both the flavedo and albedo); on the contrary, carboxylic acids are mainly found in segments and, therefore, are extracted in the juice. Regarding limonoids, they are quantitatively distributed in the albedo and segments and, consequently, are also extracted in the juice. Additionally, the composition of bioactive compounds allows for the discrimination of both citrus tissues and fruits. Our determination of these fruits’ antioxidant properties demonstrated that limonoid content can be correlated with the antioxidant activity measured by the β-carotene bleaching assay, for which there is a synergistic effect caused by other families. However, the radical scavenging activity was explained by flavonoids and ascorbic acid. Finally, these results do not enable us to draw plausible conclusions about the contributions of individual compounds to the bioactive properties of the complex extracts. However, the fractionation of the extracts provided information about the antioxidant capacity of the three main chemical families.

## Data Availability

The data presented in this study are available in this article and in the Supplementary Materials.

## Supplementary Materials

The following supporting information can be downloaded at: https://www.mdpi.com/article/10.3390/antiox12040781/s1, Figure S1: 3D PCA score plots obtained by considering all extracts grouped by fruit, tissue, and fruit × tissue. Figure S2: Relative concentrations of the monitored chemical families in the three fractions and crude extracts from citrus fruits. These results were obtained as mean results by considering the three citrus fruits in order to demonstrate their respective capacities after fractionation. Table S1: Parameters for identification of bioactive compounds in the studied citrus fruits. Table S2: Concentrations (µg/g) of flavonoids, limonoids, phenolic acids, and carboxylic acids found in citrus fruits.
