# Supplementary material for: Spatial Distribution and Antioxidant Activity of Extracts from Citrus Fruits
Source: Antioxidants (Basel). 2023 Mar 23;12(4):781. doi: 10.3390/antiox12040781 (PMC10135098; doi:10.3390/antiox12040781)
Supplement: Supplementary file 1 [file antioxidants-12-00781-s001.zip › Supplementary Table 1.pdf]

Supplementary table 1. Parameters for identification of bioactive compounds in the studied citrus fruits.

Compound name	Formula	Mass	RT	*m/z*	Deprotonated ion	Main product ions			CAS ID	
						1	2	3		
**Flavonoids**										
Naringenin	$C_{15}H_{12}O_5$	272.0680	9.76	*273.0720*	[M-H]$^{-}$	153.0186	147.0441		480-41-1	*a*
Naringenin (isomer)	$C_{15}H_{12}O_5$	272.0690	10.59	*273.0720*	[M-H]$^{-}$	153.0156	147.0400		480-41-1	*b*
Luteolin	$C_{15}H_{10}O_6$	286.0490	9.59	*287.0555*	[M-H]$^{-}$	153.0172			491-70-3	*a*
Diosmetin	$C_{16}H_{12}O_6$	300.0630	13.46	*301.0728*	[M-H]$^{-}$	286.0408	258.0527	153.0125	520-34-3	*a*
Hesperetin	$C_{16}H_{14}O_6$	302.0780	13.68	*303.0867*	[M-H]$^{-}$	177.0549	153.0185	111.0050		*a*
Tangeritin (isomer 1)	$C_{20}H_{20}O_7$	372.1200	14.26	*373.1294*	[M-H]$^{-}$	343.0869	312.1041	153.0205		*b*
Tangeritin (isomer 2)	$C_{20}H_{20}O_7$	372.1200	13.70	*373..1296*	[M-H]$^{-}$	358.1058	343.0829	153.0173		*b*
Tangeritin	$C_{20}H_{20}O_7$	372.1220	15.38	*373.1294*	[M-H]$^{-}$	358.1061	343.0826	312.1010		*a*
Vitexin	$C_{21}H_{20}O_{10}$	432.1060	9.44	*433.1154*	[M-H]$^{-}$	337.0713	313.0715	283.0609	3681-93-4	*a*
Quercetin-3-arabinoside	$C_{20}H_{18}O_{11}$	434.0970	14.32	*435.1033*	[M-H]$^{-}$	303.0498	153.0179		22255-13-6	*a*
Homoorientin	$C_{21}H_{20}O_{11}$	448.1020	8.92	*449.1095*	[M-H]$^{-}$	329.0663	299.0551	127.0382	4261-42-1	*a*
Orientin	$C_{21}H_{20}O_{11}$	448.1030	9.14	*449.1053*	[M-H]$^{-}$	329.0543	299.0445	127.0334	28608-75-5	*a*
Quercetin-3'-glucoside	$C_{21}H_{20}O_{12}$	464.0960	9.22	*465.1030*	[M-H]$^{-}$	303.0499	153.0171	129.0529	482-35-9	*a*
Hesperetin 7-O-glucoside	$C_{22}H_{24}O_{11}$	464.1318	9.97	*465.1393*	[M-H]$^{-}$	303.0871	53.0172		114248-44-1	*a*
Isorhamnetin3-O-glucoside	$C_{22}H_{22}O_{12}$	478.1130	10.25	*479.1215*	[M-H]$^{-}$	317.0669	97.0274		5041-82-7	*a*
Rhoifolin	$C_{27}H_{30}O_{14}$	578.1640	9.62	*579.1718*	[M-H]$^{-}$	433.1151	271.0611	129.0548	17306-46-6	*a*
Vitexin-2"-O-rhamnoside	$C_{27}H_{30}O_{14}$	578.1650	9.14	*579.1677*	[M-H]$^{-}$	433.1191	313.0745	283.0653	64820-99-1	*a*
Naringin	$C_{27}H_{32}O_{14}$	580.1800	8.78	*581.1877*	[M-H]$^{-}$	273.0743	195.0272	129.0537		*a*
Naringin (isomer)	$C_{27}H_{32}O_{14}$	580.1820	9.88	*581.1877*	[M-H]$^{-}$	273.0760	195.0292	129.0545	10236-47-2	*b*
Eriocitrin	$C_{27}H_{32}O_{15}$	596.1770	9.25	*597.1830*	[M-H]$^{-}$	289.0750	129.0566		13463-28-0	*a*
Diosmin	$C_{28}H_{32}O_{15}$	608.1750	9.85	*609.1833*	[M-H]$^{-}$	463.1262	301.0722	129.0544		*a*
Rutin	$C_{27}H_{30}O_{16}$	610.1540	9.21	*611.1625*	[M-H]$^{-}$	303.0516	153.0176	129.0553	153-18-4	*a*
Neohesperidin	$C_{28}H_{34}O_{15}$	610.1910	10.56	*611.1986*	[M-H]$^{-}$	303.0852	153.0174	129.0537	13241-33-3	*a*
Hesperidin	$C_{28}H_{34}O_{15}$	610.1920	10.05	*611.1986*	[M-H]$^{-}$	303.0874	129.0550	153.0178	520-26-3	*a*
Isorhamnetin-3-O-rutinoside	$C_{28}H_{32}O_{16}$	624.1650	8.66	*623.1591*	[M-H]-	315.0489	300.0262		604-80-8	*a*
Luteolin-Rha-Glu	$C_{27}H_{30}O_{15}$	594.1610	9.23	*595.1679*	[M-H]$^{-}$	449.1104	287.0564			*c*
Apigenin-Glu-Gal	$C_{27}H_{30}O_{15}$	594.1620	8.95	*595.1679*	[M-H]$^{-}$	433.1130	613.0000			*c*
Limocitrin-Hmg-Glu	$C_{29}H_{32}O_{17}$	652.1620	10.34	*651.1510*	[M-H]$^{-}$	549.1223	507.1116	345.0585		*c*
Limocitrin-Rut	$C_{29}H_{34}O_{17}$	654.1770	9.62	*653.1685*	[M-H]$^{-}$	345.0590	303.0319	199.1116		*c*
Limocitrol-Glu-Hmg	$C_{30}H_{34}O_{18}$	682.1710	10.32	*681.1635*	[M-H]$^{-}$	609.1767	537.1194	375.0663		*c*
Eriodictyol-Glu-Rha-Glu	$C_{33}H_{42}O_{20}$	758.2220	8.51	*757.2111*	[M-H]$^{-}$	595.1640	449.1053	287.0725		*c*
**Limonoids**										
Limonin	$C_{26}H_{30}O_8$	470.1940	14.31	*471.2020*	[M-H]$^{-}$	453.1906	425.1956	161.0595	1180-71-8	*a*
Nomilin	$C_{28}H_{34}O_9$	514.2200	14.62	*515.2320*	[M-H]$^{-}$	469.2240	187.0754	161.0595	1063-77-0	*a*
Nomilin (isomer)	$C_{28}H_{34}O_9$	514.2220	13.21	*515.2320*	[M-H]$^{-}$	499.2661	187.0735	161.0566	1063-77-0	*b*
Obacunone	$C_{26}H_{30}O_7$	454.1990	14.62	*455.2048*	[M-H]$^{-}$	409.2018	331.1342	161.0598	751-03-1	*a*
Nomilinic acid	$C_{28}H_{36}O_{10}$	532.2340	14.13	*533.2325*	[M-H]$^{-}$	411.2202	369.2034	161.0619		*c*
Limonoic acid D-ring lactone	$C_{26}H_{32}O_9$	488.2040	13.71	*489.2119*	[M-H]$^{-}$	471.2005	369.3169	161.0594		*c*
Limonoic acid A-ring lactone	$C_{26}H_{32}O_9$	488.2050	9.53	*489.2119*	[M-H]$^{-}$	471.1967	369.1640	161.0577		*c*
Nomilinic acid glucoside	$C_{34}H_{48}O_{16}$	712.2980	10.64	*713.3008*	[M-H]$^{-}$	551.2486	369.2028	161.0612		*c*
21-O-Methyllimonexic acid	$C_{27}H_{32}O_{10}$	516.1970	14.36	*515.2001*	[M-H]$^{-}$	480.1376	469.1934	229.1225		*c*
**Simple phenols**										
4-Hydroxybenzoic acid	$C_7H_6O_3$	138.0320	8.20	*139.0387*	[M-H]$^{-}$	121.0282	93.0327	77.0389	99-96-7	*a*
2-Coumaric acid	$C_9H_8O_3$	164.0480	11.46	*165.0521*	[M-H]$^{-}$	147.0448	119.0496	91.0539	583-17-5	*a*
Shikimic acid	$C_7H_{10}O_5$	174.0520	3.29	*173.0475*	[M-H]$^{-}$	111.0465	93.0358	73.0309	138-59-0	*a*
Ferulic acid	$C_{10}H_{10}O_4$	194.0580	8.85	*195.0623*	[M-H]$^{-}$	177.0514	145.0253	117.0307	537-98-4	*a*
Pyrogallol	$C_6H_6O_3$	126.0320	2.80	*127.0370*	[M-H]$^{-}$	109.0294	81.0332	53.0387	87-66-1	*a*

Pyrogallol (isomer)	$C_6H_6O_3$	126.0320	4.64	*127.0370*	$[M-H]^-$	109.0266	81.0316	53.0367	87-66-1	*b*
**Carboxylic acids**										
Ascorbic acid	$C_6H_8O_6$	176.0320	3.36	*175.0231*	$[M-H]^-$	115.0023	87.0079	71.0129	50-81-7	*a*
Citric acid	$C_6H_8O_7$	192.0290	3.78	*191.0224*	$[M-H]^-$	111.0104	85.0513	73.0305	77-92-9	*a*
Malic acid	$C_4H_6O_5$	134.0200	3.39	*133.0133*	$[M-H]^-$	115.0035	89.0245	71.0139	6915-15-7	*a*

RT, retention time. Identification confirmed by standard (*a*), tentative identification by MS2 spectrum similarity to the isomer (score > 90%) (*b*), tentative identification by neutral mass losses and fragmentation pattern analysis (c). Glu, glucoside; Rha, rhamnoside; Rut, rutinoside; Gal, galactoside; Hmg, hydroxymethylglutaryl.
