# Supplementary material for: Spatial Distribution and Antioxidant Activity of Extracts from Citrus Fruits
Source: Antioxidants (Basel). 2023 Mar 23;12(4):781. doi: 10.3390/antiox12040781 (PMC10135098; doi:10.3390/antiox12040781)
Supplement: Supplementary file 1 [file antioxidants-12-00781-s001.zip › Supplementary Table 2.pdf]

Supplementary Table 2. Concentration (µg/g) ± standard deviation of flavonoids, limonoids, phenolic acids and carboxylic acids found in citrus fruits (n=3).

**Flavonoids**	**Lime**				**Mandarin**				**Lemon**			
	**Flavedo**	**Albedo**	**Segments**	**Juice**	**Flavedo**	**Albedo**	**Segments**	**Juice**	**Flavedo**	**Albedo**	**Segments**	**Juice**
Apigenin-Glu-Gal	1133 ± 192	492 ± 176	647 ± 88.2	759 ± 20.5	908 ± 157	170 ± 48	151 ± 42.5	343 ± 106	597 ± 402	975 ± 105	515 ± 126	659 ± 85
Diosmetin	1 ± 0.3	0.7 ± 1.4	0.6 ± 0.3	0.4 ± 0.4	18.3 ± 1.1	0.3 ± 0.2			0.4 ± 0.5	1.7 ± 0.8	1.1 ± 0.2	1.5 ± 0.98
Diosmin	1746 ± 149	1422 ± 419	2605 ± 351	800 ± 1141	2476 ± 236	168 ± 29	111 ± 11	97 ± 3	1031 ± 681	2679 ± 337	2896 ± 327	1087 ± 194
Eriocitrin	756 ± 69	1568 ± 322	1742 ± 426	741 ± 887	199 ± 10	424 ± 53	157 ± 42	144 ± 96	194 ± 52	2178 ± 230	1956 ± 26.9	987 ± 142
Eriodictyol-Glu-Rha-Glu	7.4 ± 1.7	40.2 ± 30.3	7.9 ± 2	0.9 ± 0.1	8.99 ± 5.2	23.3 ± 1.1	10.3 ± 2	0.9 ± 0	10.6 ± 5.2	113 ± 55.7	6.4 ± 7.9	8.1 ± 2.5
Hesperetin	4 ± 1.4	1.8 ± 2.8	0.9 ± 0.3	1 ± 1.2	0.98 ± 0.3	4.8 ± 2	0.6 ± 1.1	0.3 ± 0.2	0.2 ± 0.1	1.4 ± 0.2	0.6 ± 0.3	3.1 ± 0.8
Hesperetin7-O-glucoside	34.7 ± 3.5	31.2 ± 12	38.9 ± 9.5	18.9 ± 26.8	41.4 ± 7.7	108 ± 328	5.5 ± 3.2	4.9 ± 4.8	7.6 ± 5	59.1 ± 19.9	52.5 ± 7.5	19.2 ± 2.4
Hesperidin	929 ± 361	5907 ± 418	4525 ± 552	1483 ± 1924	5069 ± 491	8366 ± 889	4424 ± 1389	2656 ± 3985	237 ± 82	6480 ± 548	3653 ± 695	2097 ± 306
Homoorientin	365 ± 41.8	17.9 ± 8.3	38.8 ± 23.7	16.6 ± 2.5	206 ± 16.1	11.2 ± 0.4	12.6 ± 0.6	12.8 ± 3.7	41.3 ± 31.9	98.5 ± 29.4	77.1 ± 18	20.6 ± 4.03
Isorhamnetin-3-O-rutinoside	1597 ± 113	650 ± 224	970 ± 180	886 ± 1219	1514 ± 163	402 ± 35.9	320 ± 62.1	291 ± 171	1314 ± 694	1339 ± 215	1704 ± 250	1970 ± 353
Limocitrin-HMG-Glu	6775 ± 914	317 ± 250	180 ± 20.8	550 ± 624	8162 ± 426	403 ± 130	153 ± 51.6	729 ± 1239	2744 ± 1615	391 ± 278	299 ± 25.8	1342 ± 25.8
Limocitrin-Rut	444 ± 64.1	34.1 ± 22.8	27.8 ± 5.1	39.4 ± 32.3	361 ± 33.8	29.4 ± 3.2	22.3 ± 3	41.5 ± 21.1	217 ± 129	30.6 ± 6	45.4 ± 6.9	104 ± 16.4
Limocitrol-Glu-HMG	3849 ± 411	183 ± 242	25.8 ± 6	149 ± 215	958 ± 60	34.5 ± 28	17.5 ± 3.9	63.7 ± 108	2000 ± 1243	238 ± 224	102 ± 15	320 ± 18.05
Luteolin	0.4 ± 0.2	0.4 ± 0.3	0.7 ± 0.4	0.3 ± 0.2	0.6 ± 0.1	6.7 ± 3.1	4.3 ± 3.6	3.5 ± 5.6		1.3 ± 0.4	1.2 ± 0.2	
Luteolin-Rha-Glu	1043 ± 48.6	589 ± 155	713 ± 154	267 ± 292	1235 ± 177	266 ± 529	99.3 ± 12.7	109 ± 32.1	460 ± 294	1423 ± 180	1192 ± 216	600 ± 115
Naringenin	1.4 ± 0.6	14.3 ± 2	5.9 ± 2.9	2.6 ± 3.7	15.9 ± 1.9	162 ± 22.4	43.4 ± 26.5	53.7 ± 82.9	0.5 ± 0.3	10.7 ± 1	1.2 ± 0.5	1.6 ± 0.1
Naringin	203 ± 9.7	1254 ± 102	717 ± 284	202 ± 151	1106 ± 111	5369 ± 352	2797 ± 1073	2151 ± 3066	138 ± 17.1	831 ± 30	259 ± 36.5	230 ± 22.6
Neohesperidin	132 ± 52.5	676 ± 1247	261 ± 27.5	128 ± 46	365 ± 38	1659 ± 3645	269 ± 56.4	280 ± 349	224 ± 57.2	617 ± 122	207 ± 33.5	157 ± 16.5
Orientin	143 ± 7.8	15.1 ± 5.3	21.6 ± 3.1	13.6 ± 2.7	121 ± 51	10.2 ± 0.5	10.4 ± 1.1	11.7 ± 1.8	11.2 ± 1	73.1 ± 19.3	41.8 ± 7.9	16.3 ± 4.8
Quercetin-3-arabinoside	10.9 ± 1.9	2.7 ± 0.8	0.9 ± 0	2.1 ± 0.8	2.5 ± 0.4	0.9 ± 0	0.9 ± 0	0.9 ± 0	11 ± 8	0.9 ± 0	0.9 ± 0	1.9 ± 0.5
Quercetin-3'-glucoside	8.8 ± 3.1	3.6 ± 2.4	3.3 ± 2.5	0.9 ± 0	41.5 ± 10.4	4.5 ± 0.6	2.8 ± 0.8	0.9 ± 0	0.9 ± 0	11.2 ± 1.6	5.8 ± 2.4	3.1 ± 1.8
Rhoifolin	233 ± 10.1	302 ± 57.1	227 ± 63	22.1 ± 8.5	196 ± 34.6	334 ± 24.6	192 ± 75.3	100 ± 189	56.3 ± 18.1	147 ± 14.4	81 ± 27	123 ± 23.9
Rutin	501 ± 48.6	16.3 ± 5.9	109 ± 47.4	108 ± 155	1280 ± 144	136 ± 45.1	238 ± 131	209 ± 367	224 ± 170	26.9 ± 2.8	188 ± 38.4	273 ± 47.1
Tangeritin	82 ± 28	1.9 ± 1.2	4.4 ± 6.1	2.3 ± 2.5	4243 ± 359	217 ± 111	23.1 ± 27.4	293 ± 556	32.95 ± 29.8	0.2 ± 0.1	0.8 ± 1.3	2.6 ± 0.7
Vitexin	367 ± 22.7	67.3 ± 25.1	33.7 ± 4.7	6.9 ± 9.8	28.7 ± 3.03	1.7 ± 0.2	1.4 ± 1.5	1.5 ± 1.02	14.3 ± 15	155.5 ± 18.7	11.8 ± 5.8	6.9 ± 0.9
Vitexin-2''-O-rhamnoside	432 ± 33.2	1184 ± 267	1076 ± 127	237 ± 235	261.4 ± 10.4	104 ± 21.2	99 ± 6.1	98.6 ± 6.2	118 ± 24.7	811 ± 194	261 ± 61.3	127 ± 8.1
Total flavonoids	20801 ± 1999	14791 ± 2308	13983 ± 1934	6439 ± 6956	28820 ± 2165	18416 ± 3218	9165 ± 3013	7697 ± 9862	9684 ± 5531	18694 ± 2274	13560 ± 1153	10163 ± 1331

**Limonoids**	**Lime**				**Mandarin**				**Lemon**			
	**Flavedo**	**Albedo**	**Segments**	**Juice**	**Flavedo**	**Albedo**	**Segments**	**Juice**	**Flavedo**	**Albedo**	**Segments**	**Juice**
Nomilinicacid		3.3 ± 2.6	13.4 ± 22.4					2.8 ± 4.4		51.5 ± 6.9	23 ± 11	14.1 ± 8.8
Nomilinicacidglucoside		2.4 ± 3.2	142 ± 88	9.3 ± 8.1			15.7 ± 12.8	67.6 ± 234		41.3 ± 13.8	209 ± 50	284 ± 49
Obacunone		5.8 ± 10.2								53.8 ± 11.8	3.2 ± 10.3	16.1 ± 4
21-O-Methyllimonexicacid	43.7 ± 12.7		105 ± 87.2	47.1 ± 63.4	4.5 ± 1.6	53.7 ± 17.2	59 ± 64	138 ± 344	48.1 ± 26.8	753 ± 72.3	260 ± 136	583 ± 69.6

Limonin	68.8 ± 20.04	27.2 ± 17.1	493 ± 182	286 ± 309	12.1 ± 1.8	92 ± 23.7	237 ± 169	554 ± 469	175 ± 90.1	1354 ± 155	960 ± 158	1007 ± 111
LimonoicacidD-ringlactone	19.5 ± 1.8	5.1 ± 5.9	22.5 ± 10.7	27.4 ± 2.2	0.5 ± 1.1	6.5 ± 4.5	18.6 ± 17	53.7 ± 104	8 ± 6	42.8 ± 20.7	31.4 ± 3.6	24.1 ± 3.5
Nomilin	11.2 ± 0.4	100 ± 100	154 ± 117	159 ± 261	3.7 ± 0.6	48.9 ± 3.2	86.3 ± 69.3	195 ± 363	101 ± 63.5	1336 ± 108	555 ± 204	536 ± 64.9
Total limonoids	143 ± 32.5	144 ± 125	929 ± 507	529 ± 503	20.9 ± 3.3	201 ± 44.9	417 ± 332	1014 ± 1438	332 ± 186	3632 ± 218	2041 ± 510	2464 ± 287
**SimplePhenols**	**Lime**				**Mandarin**				**Lemon**			
	**Flavedo**	**Albedo**	**Segments**	**Juice**	**Flavedo**	**Albedo**	**Segments**	**Juice**	**Flavedo**	**Albedo**	**Segments**	**Juice**
Shikimicacid	6.3 ± 0.6	3.9 ± 1.6	4.7 ± 1.3	2.1 ± 0.2	7.8 ± 0.3	29.5 ± 4.6	28.5 ± 7.4	7.5 ± 1.2	6.4 ± 2.8	6.4 ± 2	11.6 ± 1.6	0.7 ± 0.4
2-Coumaricacid	19.5 ± 3.6	15.3 ± 3.9	1.2 ± 1.2	1.7 ± 2			0.3 ± 0.2	1.5 ± 3.3	12.2 ± 5	60.1 ± 34.3	10.4 ± 3.4	17.3 ± 3.6
Pyrogallol	135 ± 17.2		307 ± 30.3	351 ± 77.1	413 ± 27.2	382 ± 14.6	413 ± 4.01	154 ± 100	131 ± 34.1	774 ± 81.6	285 ± 37	282 ± 54.7
Ferulicacid	3.4 ± 0.8	10.1 ± 6.2	8 ± 2.9	22.03 ± 28.1	11.7 ± 2.8	17.1 ± 3.1	10.2 ± 4.7	41 ± 65.6	10.6 ± 6.5	128 ± 33.7	40.7 ± 8.1	159 ± 24.6
4-Hydroxybenzoicacid	0.3 ± 0.3	1.1 ± 0.5	1.3 ± 0.4	2.1 ± 3.1	4.04 ± 0.3	2.8 ± 0.2	1.2 ± 0.9	2.5 ± 1.4	2.9 ± 1.4	58.2 ± 17.4	4.7 ± 0.9	10 ± 1.5
Total phenolic acids	164 ± 12.9	30.4 ± 10.4	322 ± 28.5	379 ± 47.8	437 ± 29	431 ± 9.4	453 ± 12.5	206 ± 152	163 ± 47.5	1027 ± 152	352 ± 37	469 ± 83.8
**Carboxylic acids**	**Lime**				**Mandarin**				**Lemon**			
	**Flavedo**	**Albedo**	**Segments**	**Juice**	**Flavedo**	**Albedo**	**Segments**	**Juice**	**Flavedo**	**Albedo**	**Segments**	**Juice**
Malicacid	154 ± 2.9	715 ± 363	1260 ± 491	1636 ± 477	145 ± 6.8	542 ± 85.2	818 ± 260	1325 ± 3070	138 ± 3.3	790 ± 103	1433 ± 227	7648 ± 2491
Ascorbicacid	12.5 ± 1.4	117 ± 137	266 ± 131	648 ± 799	133 ± 11.9	135 ± 76.1	556 ± 229	947 ± 1562	13.5 ± 2.1	1027 ± 114	1213 ± 238	1817 ± 280
Citricacid	141 ± 26	6017 ± 3691	12840 ± 2246	10267 ± 3238	161 ± 3.6	176 ± 19.9	3636 ± 1367	11881 ± 30451	147 ± 9.6	7713 ± 2924	15715 ± 2013	51982 ± 11520
Total carboxylic acids	307 ± 30	6849 ± 4168	14367 ± 2216	12551 ± 2945	439 ± 8.2	852 ± 173	5011 ± 1851	14153 ± 34287	298 ± 10.3	9529 ± 2971	18361 ± 2348	61447 ± 14286
