# Supplementary figures and images for: Spatial Distribution and Antioxidant Activity of Extracts from Citrus Fruits
Source: Antioxidants (Basel). 2023 Mar 23;12(4):781. doi: 10.3390/antiox12040781 (PMC10135098; doi:10.3390/antiox12040781)

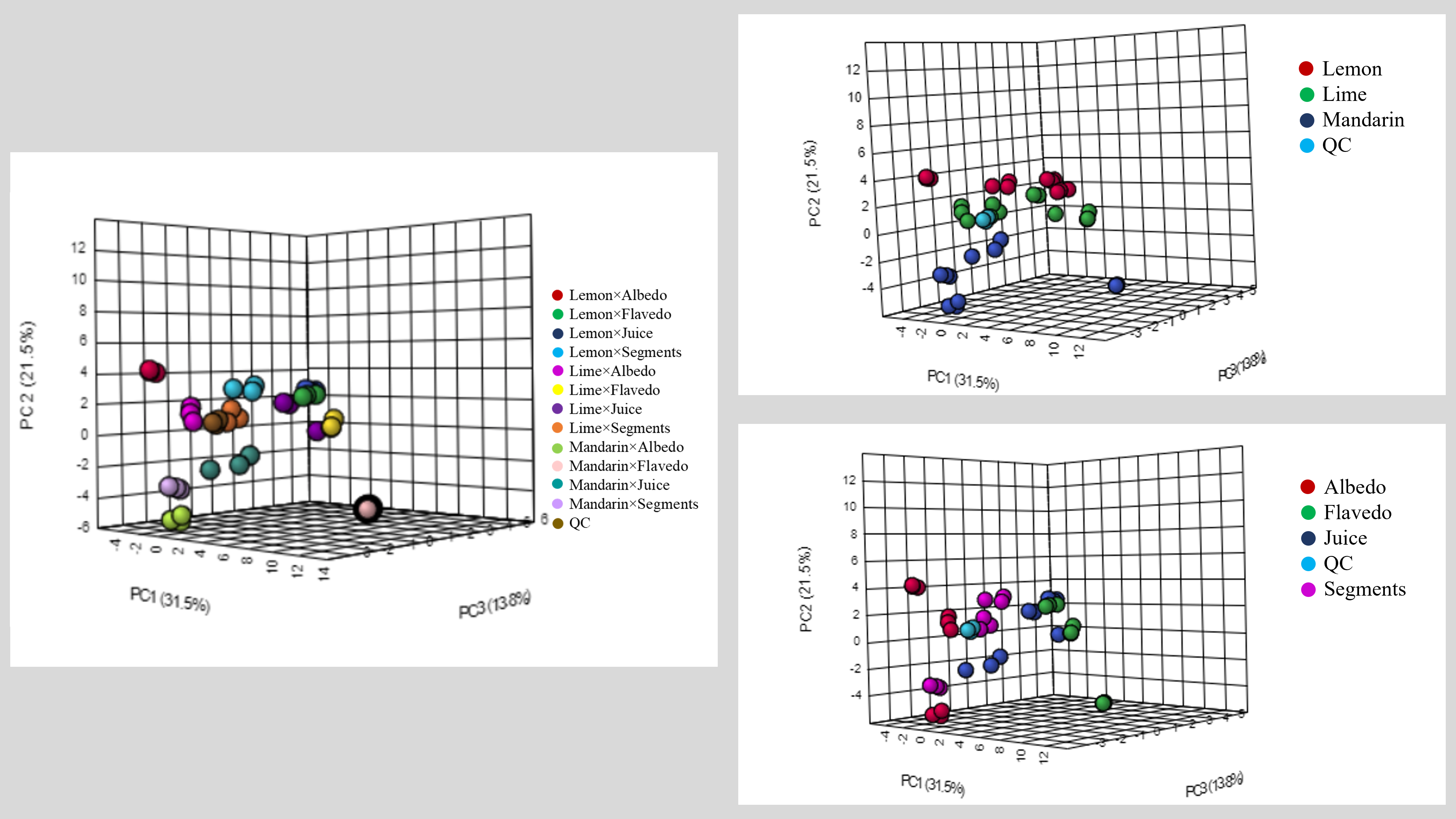

Supplement: Supplementary file 1 [file antioxidants-12-00781-s001.zip › Supplementary figure 1.png]

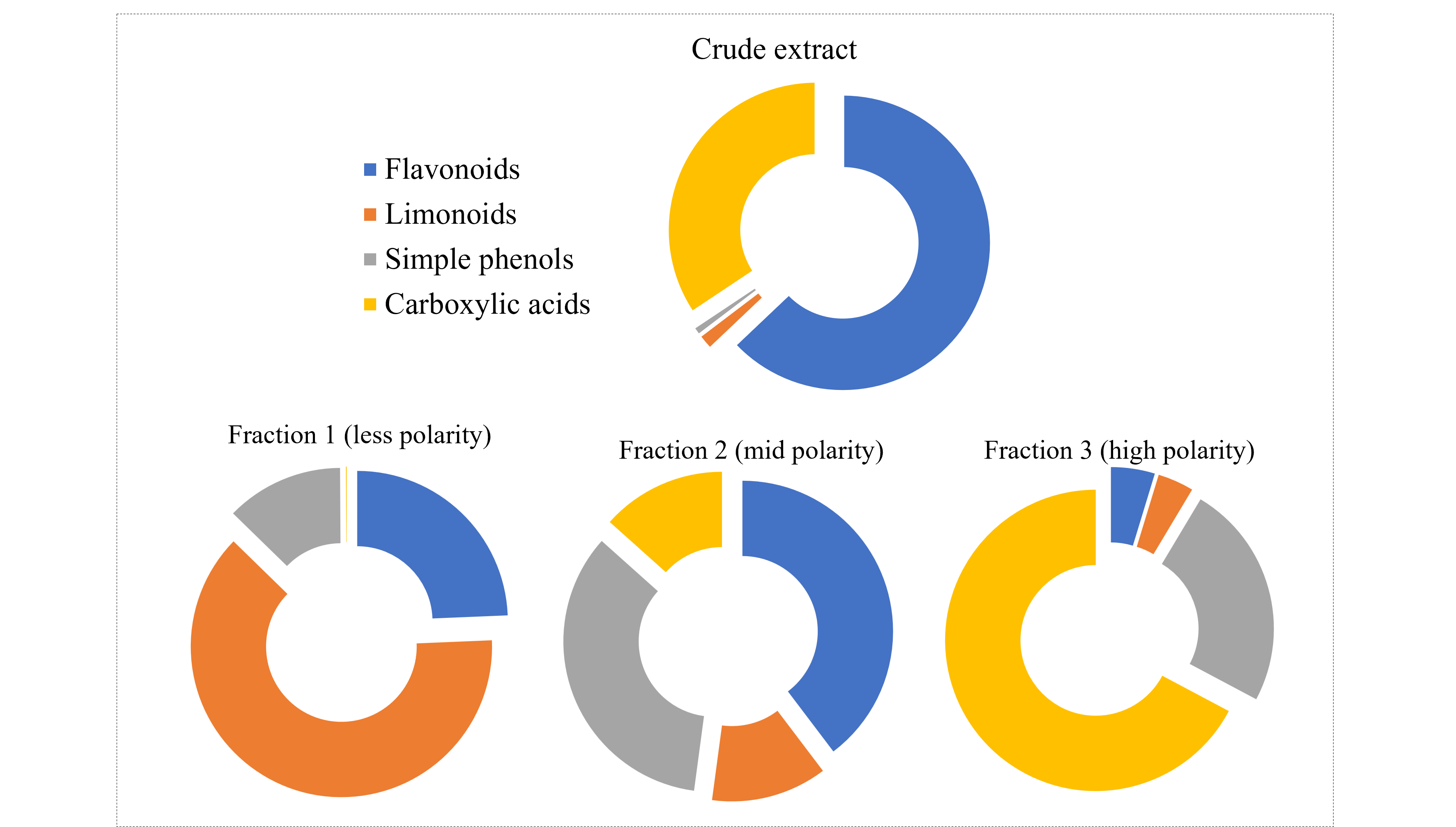

Supplement: Supplementary file 1 [file antioxidants-12-00781-s001.zip › Supplementary figure 2.png]
